# Activity based restorative therapy considerations for children: medical and therapeutic perspectives for the pediatric population

**DOI:** 10.3389/fresc.2023.1186212

**Published:** 2023-09-06

**Authors:** Brooke Reeves, Emily Smith, Miranda Broussard, Rebecca Martin

**Affiliations:** ^1^ International Center for Spinal Cord Injury, Kennedy Krieger Institute, Baltimore, MD, United States; ^2^ Department of Physical Medicine and Rehabilitation, Johns Hopkins School of Medicine, Baltimore, MD, United States

**Keywords:** spinal cord injury, activity-based therapy, pediatrics, pediatric spinal cord injury, rehabilitation

## Abstract

Well-established scientific evidence demonstrates that activity is essential for the development and repair of the central nervous system, yet traditional rehabilitation approaches target muscles only above the lesion as a means of compensation. Activity-Based Rehabilitation (ABR) represents an evolving paradigm shift in neurorehabilitation targeting activation of the neuromuscular system below the lesion. Based on activity-dependent plasticity, ABR offers high intensity activation of the nervous system to optimize the capacity for recovery, while working to offset the chronic complications that occur as a result of neurologic injury. Treatment focus shifts from compensatory training to promotion of restoration of function with special emphasis on normalizing sensory cues and movement kinematics. ABR in children carries special considerations for a developing nervous system and the focus is not just restoring functions but advancing functions in line with typical development. Application of activity-based interventions includes traditional rehabilitation strategies at higher intensity and frequency than in traditional models, including locomotor training, functional electrical stimulation, massed practice, and task specific training, applied across the continuum of care from early intervention to the chronic condition.

## Introduction

In the United States, 20% of all spinal cord injuries (SCI) occur to individual under the age of 20 (1). Traumatic SCI are more likely to occur over the age of 16 and represent 13.2 cases per million population in North America, whereas nontraumatic SCI represents 2.1 cases per million population (2). Pediatric SCI presents several factors that can contribute to treatment challenges including age, attention, motivation, ability and willingness to participate in specific activities, and communication abilities. Activity Based Rehabilitation (ABR) offers the opportunity for repeated, near normal input to assist in the recovery of functions lost to injury, or in the case of young children acquisition of developmental milestones. This dual focus is exclusive to pediatric SCI and, in combination with the unique needs of children and families, represents a shift for therapists in their treatment planning and outcomes expectations. The primary factors that need to be considered are age, the lifelong process, and parental involvement. A therapist must consider what developmental stage the child is at when planning treatment to ensure age-appropriate development. The lifelong process requires that goals change as the child does or does not meet various developmental milestones (3). Additionally, priorities and roles of the child and family change requiring modification to therapy and goals. Finally, frequent updating prevents boredom and increases motivation for child participation. Understanding parental goals while making the child feel seen and heard regarding their role is crucial.

When adults are injured, they have gone through their life participating in day-to-day activities and occupations and skills learned as children have been translated into adulthood. Young children have not met developmental milestones prior to injury impacting overall development. When a young child is injured many areas of development such as physical, social, emotional, and mental are interrupted creating challenges for their entire life (4). Therapists must keep in mind when working with children that play is a child's number one occupation, play must be what therapy is centered around to ensure participation. In this review, several case studies will be discussed illustrating the principles of ABR in the pediatric population, and the integration of play based therapy to increase compliance.

## Science and key components of ABR

Research has focused on multiple strategies to overcome the damage following spinal cord injury (SCI). This evidence includes blocking inhibitor molecules released by the glial scar and damaged myelin, administering exogenous cells into the area of damage to restore lost cells, repairing, and restoring myelin to damaged axons, encouraging axonal regrowth, and creating bridges and scaffolding across damaged areas. This scaffolding helps facilitate both tissue growth and blood flow to connect with the healthy tissue below the level of injury. This results in a way to bridge the gap above and below the level of injury as well as encourage repair of the cord and increase overall recovery (5).

While researchers have been studying procedures and medications to repair the spinal cord, strong evidence is emerging to support ABR as a mechanism for recovery (6). Combining pharmacological and therapeutic interventions may yield an even greater efficacy and recovery (6). ABR uses conventional therapeutic approaches, but at a higher frequency and intensity. There is increased focus on provided stimulation below the level of injury to optimize the nervous system for recovery and enhance the physical integrity of the body (7). This prepares the nervous system for recovery by mobilizing the endogenous cells, who work to repair and remyelinate (8). Interventions such as functional electrical stimulation, range of motion, and weight bearing all aid in maintaining bone mass, muscle health, and tendon length while offsetting the rapid aging and deterioration that people with spinal cord injury experience. This results in less complications, improved life expectancy and increased quality of life. It also limits long term expenses by allowing people to return to work, participate in their preferred leisure and social activities, and reduces caregiver burden (4).

ABR is repeated, near- normal activity, above and below the level of the lesion. This therapy is intended to optimize the neurological system and reduce or reverse the physical deterioration, secondary complications, and rapid aging associated with SCI (9). This rehabilitation is characterized by high intensity, task specific, and pattered activity below the level of injury. The goal of ABR is to restore central nervous system function and promote neural recovery and regeneration. Combining high intensity practice with non- patterned and patterned movements restores lost function, aiming to near normal kinematics and minimizing or eliminating compensatory devices. Comparatively, traditional therapy preferentially actives the nervous system above the level of injury and uses low intensity practice with non-patterned movements (10). This compensates for loss in function by using compensatory devices rather than restoring function. ABR provides regular, routine exercise thus increasing overall quality of life and preventing chronic diseases (9).

The following key components make up ABR: functional electrical stimulation (FES), locomotor training (LT), weight bearing, massed practice, task- specific practice, aquatic therapy, and home-based rehabilitation programs.

There are three categories of electrical stimulation most utilized in ABR which include neuromuscular electrical stimulation (NMES), FES, and transcutaneous electrical nerve stimulation (TENS). NMES and FES both apply electrical pulses across the skin, evoking an action potential and causing muscle contraction. FES is often paired with task- specific practice, as evidence shows that increased instance of FES use paired with increased frequency and intensity of exercise yields decreased atrophy and increased bone mass (11). TENS can be used for pain modulation by means of peripheral nerve stimulation, and uses sensory, motor, or noxious settings. Sensory settings utilized in TENS are also effective in providing sensory input to the nervous system for tone and spasticity management. These settings are most effective when paired with rigorous activity, as a study found that combined leads to improvements in tone, functional activity, and gait efficiency (12). Electrical stimulation may prevent or reverse disuse atrophy, as well as improve and maintain muscle mass and range of motion. Stimulation maintains bone health, improves cardiovascular status, normalizes tone, and optimizes the nervous system for recovery (11).

Weight bearing promotes joint alignment, bone stress, and muscle co-contraction, while also promoting normalized afferent input. Weight bearing can be achieved through the upper extremities, and lower extremities through prone positioning, propping, quadruped, kneeling, and standing. Weight bearing is a crucial part of development increasing control and stability of the joints, as well as progressing overall mobility and cognition. Pairing weight bearing with patterned functional movement improves bladder function and bowel motility, decreases pressure injuries, improves range of motion, decreases spasticity, improves bone health, thus improving overall quality of life (13).

Locomotor training is a strategy designed to improve sensory, motor, and autonomic function, health and quality of life. Sensory cues retrain neural patterns, resulting in effective locomotion. This emphasizes recovery of motor function, rather than compensatory strategies, by using the intrinsic mechanisms of the nervous system (14). Locomotor training can improve walking speed, independence, and endurance, as well as balance, motor recovery gross motor skills, and well- being (13, 15, 16). Combined with weight bearing through the legs, locomotor training optimizes the sensory cues and kinematics for motor tasks. Locomotor training can be done overground, on a treadmill, and through community training (14).

Massed practice includes repetitive, task specific and non- task specific activities and promotes cortical reorganization. Studies show that the combination of long- term therapy and repetitive practice yields positive results for motor learning and recovery (17). According to Jackman et al. (18), children need approximately 40 h of practice to obtain clinically meaningful change of general upper limb function. Traditional rehabilitation does not adequately drive neural reorganization needed to promote optimal function.

Task specific practice includes specific motor tasks aimed at functional training rather than adapting to an impairment. This practice is goal directed, and most successful when paired with feedback and facilitation via NMES and EMG. Task specific practice incorporates five major principles: relevant, random, repetitive, reconstruction, and reinforced. A study focused on task specific balance training in children with CP showed that there was an overall improvement in stability compared to the control group (19). Positive outcomes are yielded when large amounts of therapy time are paired with functional activities and remediation.

ABR principles can be applied in the aquatic environment to facilitate weaker muscle groups and provide resistance against stronger muscle groups. Properties of water including buoyancy, viscosity, turbulence, hydrostatic pressure, and warmth can provide strengthening, tone management, and sensory input. Home rehabilitation programs offer patients a long-term guide to incorporating ABR principles into their daily routines. These programs focus on patient's impairments and functional limitations and incorporate things like FES, weight bearing, and massed practice (20). Education to families and caregivers is also provided to ensure continuation of therapy and care following discharge.

## New or old: rehabilitation vs. habilitation

The importance of considering the age the child was injured and where they were at in their development will determine whether their treatment is rehabilitative or habilitative. Rehabilitation is the restoration of health through training or therapy after illness or injury (21). This includes improving strength in spared muscles and teaching alternative compensatory patterns to restore optimal functional independence to substitute for neurological deficits. Rehabilitation helps to prevent complications and help individuals adapt to an altered lifestyle resulting from loss of function. Habilitation is teaching or guiding children through development of mobility, orientation and independence in skills as they prepare for adulthood (22). These techniques are practiced and incorporated into everyday situations, adapting and evolving as the child progresses through developmental stages (22). Children rarely understand the end goal, as they have never been there before, so they do not have old learning and skills to fall back on. With habilitation, goals are always changing, and it is very easy for them to fall behind their peers. Key things to remember when utilizing habilitation is to meet the child where they are at developmentally, don't jump right to highly skilled intervention. Incorporation of age-appropriate play through games, activities, and similar aged peers assists in motivation for children, as play is the hallmark of child development.

Goal development is an integral part of the plan of care and plays a major role in how to integrate ABR into treatment sessions. There are several principles to incorporate into goal development including: activation of the nervous system above and below the level of injury, provide independent mobility, facilitation of developmental milestones, help children achieve their maximal potential and full integration as a productive member of society. Prioritizing the goals of the child and family is important for compliance and therapy buy-in, as well as modification of the plan of care as the child progresses.

The plasticity inherent in a developing nervous system may contribute to improved outcomes. Neuroplasticity involves adaptive structural and functional changes in response to activity related stimuli. Owing to its focus on high volume repetition of near normal activities, ABR aims to engages the nervous system's capacity for change to improve function (23).

Not only does neuroplasticity contribute to recovery and participation, developmental stages and the age of the child impact treatment. Treatments should be planned according to the milestones a child should be hitting. Additionally, one should take into consideration the amount of independence a child has at any given stage or the amount the child relies on from their parents. Treatment planning for an infant is going to look different than treatment planning and the plan of care for a school aged child. While the physical age of the child needs to be considered, one must also consider where the child falls in terms of emotional, mental, and social development. A therapist would not treat a school aged child who presents with the cognition of a toddler the same as a school aged child who presents with the cognition of a school aged child.

Applying ABR into treatment sessions may differ depending on age and what equipment is available to you. Always consider where the child should be developmentally, however you will have to start with the milestones they have not yet achieved. With infants, it is important to start slowly and build rapport, use parents to your advantage by having them participate in the session. With all children, we must simulate activities so that they contribute to age-appropriate development and milestones; incorporating as many developmental positions as possible. For example, weight bearing through forearms prior to quadruped aids in dissociation and development of the shoulder girdle, which helps with spine extension. This also increases lower extremity control and therefore lower extremity dissociation in preparation for quadruped. Quadruped then increases pelvic control, increasing stability, eventually leading to creeping which is essential for cognitive development.

## Clinical application of activity based rehabilitation

ABR principles are applied during intervention with specific goals. FES can be combined with functional activities with a portable unit and triggered stimulation, as an orthotic substitution, FES cycling, or as biofeedback. Weightbearing can be achieved via static or dynamic standers, supported by bracing, and with assistance as needed. Locomotor training is applied through three components: treadmill training with body weight support (BWS), overground training with as much weightbearing as possible, and community training involving varying surfaces, speeds, and obstacles. Massed practice can be incorporated into other ABR components, breaking down functional skills, and utilizing proprioceptive neuromuscular facilitation (PNF). Task specific practice includes any relevant skills practiced in the following ways: random, repetitive, reconstructed, and reinforced with positive feedback.

Discussed below are several case studies involving ABR in the pediatric SCI population. Each of these individuals are at greater risk for osteoporosis and fragility fracture, hip subluxation, and neuromuscular scoliosis due to their injury. They presented with the following upon initial assessment: disuse muscle atrophy, lower extremity paralysis, impaired sensation, tone abnormalities, and inability to bear weight in lower extremities.

A fourteen-month-old male with a history of non-traumatic spinal cord injury related to spinal cord ischemia at birth is diagnosed with incomplete paraplegia. Upon initial assessment, he presented with significant lumbar kyphosis of 77 degrees, moderate to maximal assistance needed for all bed mobility, dependent assistance for maintenance of development positioning, and inability to sit without upper extremity support. Formal manual muscle testing was not performed due to age, however, no voluntary contractions were observed in the legs. The patient participated in an eight-month bout of ABR, two days per week for two hours, which consisted of all aspects of ABR, including the utilization of supported standing devices, overground standing, quadruped, and tall kneeling, all incorporated during play. Massed and task specific practice were incorporated during dynamic sitting balance, sit to stands, and floor mobility. He participated in locomotor training each week, aquatics bi-weekly, and FES in daily sessions. FES was modified for age, adjusting parameters, using smaller electrodes, decorating electrodes, and placing them on parents or toys to promote accommodation to this intervention. Therapists utilized a habilitative approach, meeting the child where he was at on a mobility level, promoting normal kinematics, and building on skills as he progressed through the stages of development.

At discharge, the patient presented with hip flexor, gluteal, and quadriceps activation against gravity, and improved trunk strength and sitting balance, playing a role in the improvement of his lumbar kyphosis to 28 degrees. He achieved independence with bed mobility, creeping with minimal assistance for lower extremity advancement, and standing with unilateral upper extremity support. He was ambulating with bilateral ankle- foot orthoses (AFOs), posterior walker, and minimal physical assistance. During his second bout of care at 2 years, 2 months old, he was ambulating with bilateral loft strand crutches and minimal assistance and was able to complete sit to stand and floor to stand transitions with minimal assistance.

This case study illustrates the developmental progression over a long-term bout of ABR and the impact of intensive early intervention for long term success of acquiring developmentally appropriate skills. Compared to traditional therapy, which offers a more limited frequency and intensity of therapy, focuses on compensatory skills, and limits number of repetition and massed practice, outcomes and progress would have likely been slowed and limited the child's functional level. These differences demonstrate that traditional therapy is insufficient to drive change compared to ABR (9).

A nineteen-year-old male diagnosed with a traumatic spinal cord injury resulting from a motor vehicle collision 15 months ago is diagnosed as C4 America Spinal Injury Association Impairment Scale Classification (AIS) A Tetraplegia and was ventilator dependent at initial evaluation on inpatient. He presented with 0/5 lower extremity activation, and upper extremity activation consisting of 3/5 shoulder flexion, 2/5 shoulder abduction, 3/5 elbow flexion, and 1/5 elbow extension. He required dependent assistance for static sitting balance, all mobility and activities of daily living (ADLs). He participated in 8 weeks of inpatient ABR with five hours of therapy per day consisting of FES cycling for upper and lower extremities, supported standing, static and dynamic sitting balance training, massed practice mobility training, prone positioning, and task specific training for ADLs. At discharge from inpatient, he was breathing independently, required minimal assistance for sitting balance, maximal assistance for rolling and transfers, improved tolerance to upright positioning, and improved independence negotiating his environment via power wheelchair. The progress above demonstrates the impact of intensive therapy immediately following an injury. Outpatient therapy continued the use of intensive therapies using ABR interventions, progressing to standing overground in harnesses with use of FES, treadmill-based locomotor training, dynamic sitting balance, and use of adaptive equipment for ADLs. At discharge from outpatient, he had developed trace contractions in bilateral hip flexors, left hip extensors, abductors, knee flexors, right adductors, and knee extensors. He was able to sit with supervision for up to 5 min and complete dynamic sitting balance activities with minimal assistance, including reaching during ADLs, and complete self-feeding tasks with an adaptive device. These outcomes demonstrate how the use of ABR principles, specifically integration of interventions above and below the level of injury positively impact function and neuro recovery. With a traditional therapy model, interventions would have likely focused on compensatory strategies and neglected activation of the nervous system below his level of injury. ABR principles optimize recovery in acute and chronic patients, by allowing for the experience and repetition to drive changes both above and below level of lesion.

## Conclusion

Capitalizing on the plasticity inherent in the developing nervous system, children have the capacity for a robust recovery following spinal cord injury. As in the typically developing nervous system, neural change is driven by experience. Traditional models of rehabilitation lack sufficient intensity to cause change (24). ABR is appropriate for a wide range of patients, both acute and chronic, and it includes interventions for all ages. ABR is vital to recovery post injury, and utilizing principles including FES, weight bearing, locomotor training, massed and task specific practice to activate the nervous system below the level of injury and optimize the nervous system for recovery. Working closely with the child, the family, as well as the other providers plays a major role in goal development, participation, and progress that is made during therapy. Special consideration should be paid to the child's development on a physical, social, emotional, and mental level at the time of their injury or illness. Time taken to build rapport, engage in play, and develop behavior modifications when appropriate will promote buy-in and compliance to maximize recovery and independence.

## Data Availability

The original contributions presented in the study are included in the article/Supplementary Material, further inquiries can be directed to the corresponding author.
